# Identification of a Metabolizing Enzyme in Human Kidney by Proteomic Correlation Profiling[Fn FN1]

**DOI:** 10.1074/mcp.M112.023853

**Published:** 2013-05-14

**Authors:** Hidetaka Sakurai, Kazuishi Kubota, Shin-ichi Inaba, Kaoru Takanaka, Akira Shinagawa

**Affiliations:** From the ‡Discovery Science and Technology Department, Daiichi Sankyo RD Novare Co., Ltd., 1-16-13, Kitakasai, Edogawa-ku, Tokyo 134-8630, Japan;; §Drug Metabolism & Pharmacokinetics Research Laboratories, Daiichi Sankyo Co., Ltd., 1-2-58, Hiromachi, Shinagawa-ku, Tokyo 140-8710, Japan

## Abstract

Molecular identification of endogenous enzymes and biologically active substances from complex biological sources remains a challenging task, and although traditional biochemical purification is sometimes regarded as outdated, it remains one of the most powerful methodologies for this purpose. While biochemical purification usually requires large amounts of starting material and many separation steps, we developed an advanced method named “proteomic correlation profiling” in our previous study. In proteomic correlation profiling, we first fractionated biological material by column chromatography, and then calculated each protein's correlation coefficient between the enzyme activity profile and protein abundance profile determined by proteomics technology toward fractions. Thereafter, we could choose possible candidates for the enzyme among proteins with a high correlation value by domain predictions using informatics tools. Ultimately, this streamlined procedure requires fewer purification steps and reduces starting materials dramatically due to low required purity compared with conventional approaches. To demonstrate the generality of this approach, we have now applied an improved workflow of proteomic correlation profiling to a drug metabolizing enzyme and successfully identified alkaline phosphatase, tissue-nonspecific isozyme (ALPL) as a phosphatase of CS-0777 phosphate (CS-0777-P), a selective sphingosine 1-phosphate receptor 1 modulator with potential benefits in the treatment of autoimmune diseases including multiple sclerosis, from human kidney extract. We identified ALPL as a candidate protein only by the 200-fold purification and only from 1 g of human kidney. The identification of ALPL as CS-0777-P phosphatase was strongly supported by a recombinant protein, and contribution of the enzyme in human kidney extract was validated by immunodepletion and a specific inhibitor. This approach can be applied to any kind of enzyme class and biologically active substance; therefore, we believe that we have provided a fast and practical option by combination of traditional biochemistry and state-of-the-art proteomic technology.

Molecular identification for an enzyme reaction or biologically active substance in an organism is challenging, although molecular biological methodologies such as expression cloning (1), recombinant protein panel (2) and RNAi screening (3) have been introduced recently as alternative approaches. Conventional biochemical purification has provided a number of successes and thus still remains a powerful, though labor-intensive strategy.

In the traditional protein purification, it had been necessary to purify an individual protein nearly to homogeneity at a microgram amount so that the purified protein could be analyzed by N-terminal amino acid sequencing. Protein identification by mass spectrometry subsequently revolutionized this technology by enabling identification of proteins at much lower abundances: individual proteins could then be associated with specific activities as soon as a band in SDS-PAGE could be observed, even when the purified protein was far from homogeneity (4–6). Although this streamlined the workflow by reducing the required starting materials as well as the separation steps for protein purification, a faster and more generalized approach from smaller starting material has still been desired because some proteins are physiochemically difficult *for example* in solubilization and stability. To solve these problems, we devised a proteomic correlation profiling methodology (7).

The basic concept of proteomic correlation profiling was originally developed by Andersen *et al.* (8). They quantitatively profiled hundreds of proteins across several centrifugation fractions by mass spectrometry and identified centrosomal proteins by calculating the correlation of these protein expression profiles with already known centrosomal proteins. In the following study, Foster *et al.* applied this strategy to map more than 1400 proteins to ten subcellular locations (9). Although these studies used centrifugation as a separation method and a known marker profile as a standard for correlation, we extended this concept to use chromatography as a separation method and kinase activity as a basis for comparison; our approach successfully identified a kinase responsible for phosphorylation of peptide substrates just after one step chromatography, and was termed proteomic correlation profiling (7). Independently, Kuromitsu *et al.* reported identification of an active substance in the serum response element-dependent luciferase assay from interstitial cystitis urine after three-step chromatography by a similar concept (10). In theory, this general proteomic correlation profiling strategy can be adapted to any kind of separation method and activity profile but no other example has been reported thus far, therefore, actual examples where the method can be applied to other enzyme classes are required to prove its generality.

Multiple sclerosis is the most common autoimmune disorder of the central nerve system in which the fatty myelin sheaths around the axons of the brain and spinal cord are damaged, leading to demyelination and scarring (11, 12). Until recently, the standard treatments for multiple sclerosis such as interferon beta, glatiramer acetate, mitoxantrone, and natalizumab would often cause severe adverse events (13, 14), providing an opportunity for development of less dangerous treatments for this disease. However, in 2010, Food and Drug Administration approved fingolimod (Gilenya; chemical structure in Fig. 1) as the first oral medicine, and recommended this as a first-line treatment for relapsing-remitting multiple sclerosis, opening up a new therapeutic approach to the disease (15).

Sphingosine 1-phosphate receptor 1 (S1P1)^1^ modulators are emerging as a new class of drugs with potential therapeutic application in multiple sclerosis (15), and fingolimod is a nonselective sphingosine 1-phosphate (S1P) receptor modulator (16–18, 21, 22). Given its structural similarity to sphingosine, fingolimod is phosphorylated *in vivo* by sphingosine kinase, in particular sphingosine kinase 2 (SPHK2) (19, 20), and the fingolimod-phosphate (fingolimod-P, Fig. 1) binds to and activates four G protein-coupled S1P receptors (21, 22). By this mechanism, fingolimod-P induces internalization of S1P1 on lymphocytes, blocking the ability of the receptor to support lymphocyte egress and recirculation through secondary lymphoid organs. This suppresses immune responses and is presumably the main immunomodulatory mode of action of fingolimod.

CS-0777 (Fig. 1) is a novel selective S1P1 modulator (23). Although the immunomodulatory effects are supposed to be mainly mediated by S1P1, some lines of evidence suggest that the agonist activity on S1P receptor 3 (S1P3) could cause acute toxicity and cardiovascular deregulation, including bradycardia in rodents (24, 25). Thus, CS-0777 was designed to have more selectivity on S1P1 over S1P3 in contrast to fingolimod-P which has potent agonistic activity for S1P3, S1P4, and S1P5 *in vitro* (22). Like fingolimod, CS-0777 is also a prodrug phosphorylated *in vivo*, and the phosphorylated CS-0777 (CS-0777-P, Fig. 1) agonizes S1P1 with more than 300-fold selectivity relative to S1P3 whereas CS-0777-P has weaker effects on S1P5 and no activity on S1P2 (23). CS-0777 showed immunosuppressive activity in mouse and rat models of experimental autoimmune encephalitis, animal models for multiple sclerosis. In healthy volunteers, single oral doses of CS-0777 caused marked, dose-dependent decreases in numbers of circulating lymphocytes, including marked and reversible decreases in circulating T and B cells (26). Furthermore, in multiple sclerosis patients, single oral doses of CS-0777 caused dose-dependent decreases in circulating lymphocytes, with a slightly greater suppression of CD4+ *versus* CD8+ T cells. Therefore, CS-0777 would alter immune responses solely through activation of S1P1 without S1P3 modulation in humans, which could circumvent a bradycardia adverse effect, although the relationships associating selectivity of S1P1 to S1P3 with bradycardia in humans are not fully understood (12).

Orally administrated CS-0777 is phosphorylated and rapidly reaches equilibrium with CS-0777-P as in the case of fingolimod (22), suggesting that the high kinase activity in blood is balanced by phosphatases. Therefore, identification of a phosphatase, the inactivating enzyme of an active metabolite, as well as identification of a kinase, the activating enzyme of a prodrug, are critical to fully understand the mechanism of action at the molecular level for both CS-0777 and fingolimod. Sphingosine kinase 2 (SPHK2) was identified as the major kinase of fingolimod (21, 28, 29) and lipid phosphate phosphatase 3 (LPP3) was reported to be a phosphatase for fingolimod-P dephosphorylation (30), although contribution of LPP3 *in vivo* has not been fully studied. In our previous work, we have identified CS-0777 kinases in human blood as fructosamine 3-kinase-related protein (FN3K-RP) and fructosamine 3-kinase (FN3K) (6), whereas the phosphatase of CS-0777-P had not been identified thus far.

In this study, we have successfully identified alkaline phosphatase, tissue-nonspecific isozyme (ALPL) as the major CS-0777-P phosphatase candidate in the human kidney by proteomic correlation profiling. According to available information, this is the first report applying proteomic correlation profiling to enzyme classes other than kinases; similarly, we believe this to be first application of proteomic correlation profiling to human tissue extract, which therefore has opened up wide usage of proteomic correlation profiling for all types of enzyme identification.

## EXPERIMENTAL PROCEDURES

### 

#### 

##### Chemicals

**Fig. 1.**
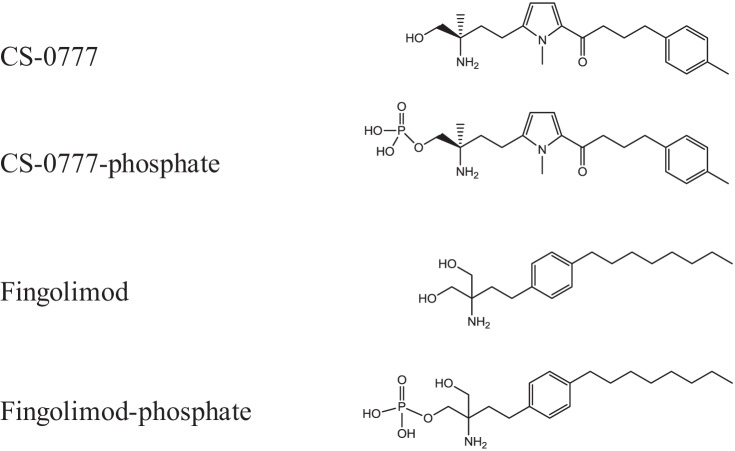
**The chemical structures of CS-0777, fingolimod and their phosphorylated derivatives.**

All chemical compounds were synthesized in Daiichi Sankyo Co., Ltd. Chemical structures are illustrated in Fig. 1.

##### Human specimens

Human frozen tissue blocks (kidney, liver, and lung) were collected by National Disease Research Interchange, and provided by the Human Animal Bridging Research Organization. The human small intestines were provided by Xenotech. Ethical approval was obtained from the Research Ethics Committee at Daiichi Sankyo Co., Ltd.

##### Purification of Human Kidney CS-0777-P Phosphatase

All purification procedures were conducted at 4 °C. The block of human kidney was minced and homogenized in 9 volumes of homogenate buffer, 0.25 m sucrose containing 4-(2-hydroxyethyl)-1-piperazineethanesulfonic acid (HEPES), pH 7.0, and protease inhibitor mixture (Complete Tablet, Roche Applied Science) using a polytron homogenizer (Kinematica). The homogenate was sonicated by a Bioruptor (COSMO BIO) and filtered by a few sheets of gauze. The tissue homogenate was centrifuged with 9000 × *g* for 60 min at 4 °C and the supernatant was used as S9 fraction.

The homogenate was centrifuged at 100,000 × *g* for 60 min, and the precipitate equivalent to 1.1 g of human kidney was dissolved in 10 ml of homogenate buffer containing 1% n-Dodecyl-β-d-maltopyranoside (DM), and then dialyzed against 1 L of anion exchange chromatography (AEX) buffer (50 mm Tris-HCl, pH 9.0, containing 0.1% DM, 5 mm CaCl_2_, 5 mm MgCl_2_, and 1 mm dithiotreitol). The dialyzed sample was filtered and loaded onto a multimodal anion exchange column (HiTrap Capto Adhere 5 ml, GE Healthcare). The column was equilibrated with AEX buffer over 30 min at a flow rate of 5 ml/min and the bound proteins were eluted with a linear gradient of 0–500 mm NaCl in AEX buffer for 10 min. The active fractions (10 ml) were dialyzed against 1 L of AEX buffer. The dialyzed sample was loaded onto an anion exchange column (Mono Q 5/50 GL, GE Healthcare). The column was equilibrated with AEX buffer over 30 min at a flow rate of 1 ml/min. The bound proteins were eluted with a linear gradient of 0–250 mm NaCl in AEX buffer for 20 min. The active fraction (1 ml) from the Mono Q column was loaded onto a gel filtration column (GFC, Superdex 200 5/50 GL, GE Healthcare). The column was equilibrated with 20 mm HEPES (pH 7.0) containing 0.1% DM, 500 mm NaCl, and 1 mm dithiotreitol (DTT) over 12 h. The separation was performed by an isocratic elution with the same buffer over 30 min at a flow rate of 40 μl/min.

##### Protein Identification and Quantification by Mass Spectrometry

Five hundred fmol of bovine serum albumin (BSA) was added as the internal standard to the fractions from the gel filtration chromatography, and then the proteins were precipitated by methanol chloroform precipitation to remove the detergent and salts (31). Briefly, four volumes of methanol, one volume of chloroform, and three volumes of water were serially added and mixed vigorously to the fractions. After centrifugation, the aqueous phase was removed and four volumes of methanol were added. The precipitated proteins were collected by centrifugation and dried completely with a centrifuge evaporator. The dried proteins were dissolved with 8 m urea, 50 mm Tris-HCl, pH 8.0, 10 mm EDTA, pH 8.0, and 0.005% DM, and 10 mm DTT. The proteins were reduced at 37 °C for 20 min, followed by alkylation by incubation at 25 °C for 20 min in the dark with 20 mm iodoacetamide. The proteins were digested with 500 ng of trypsin (Modified trypsin, Promega, Madison, WI) at 37 °C for 12 h. The reaction was stopped by acidification with 5% formic acid to a pH lower than 2.5. Samples were desalted and concentrated by using slightly modified Stage Tips (32) protocol.

The LTQ-Orbitrap (Thermo Fisher Scientific) was equipped with an Agilent 1100 liquid chromatography system, which was modified to have a 200–300 nL/min flow rate by an in-house flow splitter. A homemade electrospray ionization tip column (100 μm internal diameter x 150 mm length) was packed with Inertsil ODS-3 C_18_ (3 μm, GL Sciences). Four microliters of the sample was injected to the LC-MS/MS system, and elution of peptides was carried out with a linear gradient of acetonitrile in 0.05% formic acid. Protein identification and quantitation were performed by the MaxQuant (version 1.1.1.2, Max Planck Institute) (33–35). Detailed MaxQuant parameters are described in supplemental Table S2. Briefly, MS/MS spectra were searched against the IPI human database released on 19 Jun 2010 (in total, 89,652 protein entries) (35). The identified proteins were quantified in an ion intensity-based label-free algorithm. Two missed cleavages were allowed, along with carbamidomethylation of cysteine as a fixed modification; variable modifications were oxidation of methionine and acetylation of N-term amino acid of the protein. Mass tolerance for precursor ions was 7 ppm, mass tolerance for fragment ions was 0.5 Da, and false discovery rates at peptide and protein levels were both 0.01. The proteins identified by more than one single peptide were used for further correlation analysis.

##### Dephosphorylation Assay of CS-0777-P and Fingolimod-P

For confirmation of the CS-0777-P phosphatase activity in human kidney samples during the purification process, in gene expression experiments of candidate proteins for CS-0777-P phosphatase and in human kidney extract after immunodepletion of ALPL, the sample was incubated at 37 °C for 30 min with 10 μm CS-0777-P or 10 μm fingolimod-P in 100 mM HEPES, pH 7.0, containing 200 μg/ml BSA, 1 mm DTT, 5 mm CaCl_2_, and 5 mm MgCl_2_. After incubation, the reaction was quenched by mixing with 2 volumes of 90% methanol/0.1% acetic acid/0.077% ammonium acetate, and 5 μl of the sample was injected in the liquid chromatography equipped with a mass spectrometry (LC-MS) system. Chromatographic separation took place on a reversed-phase chromatography column (Zorbax XDB-C18 1.8 μm, 4.6 mm internal diameter × 50 mm length) and the analytes were analyzed with isocratic elution of methanol in 0.1% formic acid and 0.077% ammonium acetate. The flow rate employed was 0.5 ml/min and the column temperature was set at 50 °C. Eluting analytes were detected by selected ion monitoring on a single-quadrupole mass spectrometer (1100 LC/MSD, Agilent technologies) equipped with an electrospray ionization source and operated in positive mode. For confirmation of the inhibitory effect of an ALPL specific inhibitor on CS-0777-P and fingolimod-P phosphatase in a human kidney, the sample was incubated at 37 °C for 5 min with 1 μm CS-0777-P or 1 μm fingolimod-P in 100 mm HEPES, pH 7.4, containing 200 μg/ml BSA, 1 mm DTT, 5 mm CaCl_2_, and 5 mm MgCl_2_. After incubation, the reaction was quenched by mixing with an equal volume of 0.1% phosphoric acid/99% methanol, and 10 μl of the sample was separated by liquid chromatography followed by online tandem mass spectrometric analysis. Chromatographic separation took place on a reversed-phase chromatography column (TSK-GEL ODS-100V 5 μm, 2 mm internal diameter × 100 mm length) and the analytes were analyzed with linear gradient elution of methanol in 1% formic acid and 0.077% ammonium acetate. The flow rate employed was 0.2 ml/min and the column temperature was set at 50 °C. Eluting analytes were detected by a triple quadrupole mass spectrometer (API 5000, AB Sciex) equipped with an electrospray ionization source operated in positive mode. Product ions for CS-0777, CS-0777-P, fingolimod, and fingolimod-P were detected via multiple reaction monitoring.

Levamisole was used as an ALPL-specific inhibitor (36, 37), which was added to the sample before CS-0777-P addition to final concentrations ranging from 0–10 mm in the reaction mixture. One unit of CS-0777-P phosphatase activity was defined as the activity required for 1 ng/ml production of CS-0777 under the experimental conditions described above.

##### Proteomic Correlation Profiling

Following MaxQuant analysis, all quantified proteins within each fraction were normalized by an amount of BSA to account for any experimental error and correct for any variations in sample complexity. Each protein's normalized abundance profile was then compared against CS-0777-P phosphatase activity within each fraction while using the Pearson correlation coefficient as a measure of similarity.

##### Protein Assay

The total protein concentration was determined by modified Bradford protein assay (Coomassie Plus Protein Assay, Thermo Fisher Scientific) or Lowry protein assay (RC DC Protein Assay Kit I, Bio-Rad Laboratories, Hercules, CA) using BSA as a standard protein. Chromatographic fractions were assayed before spike-in of BSA internal standard for label-free quantitation.

##### Recombinant Protein Preparation of Candidate Proteins for CS-0777-P Phosphatase

Entry clones of ALPL (clone number: FLJ93059AAAN), alkaline phosphatase, intestinal (ALPI, FLJ93658AAAF), alkaline phosphatase, placental (ALPP, FLJ91117AAAF), Lipid phosphate phosphohydrolase 1 (LPP1, FLJ82762AAAF), lipid phosphate phosphohydrolase 2 (LPP2, FLJ82497AAAF), lipid phosphate phosphohydrolase 3 (LPP3, FLJ92787AAAF), sphingosine-1-phosphate phosphatase 1 (SPP1, FLJ94912AAAF), and sphingosine-1-phosphate phosphatase 2 (SPP2, FLJ95081AAAF) were obtained from the National Institute of Technology and Evaluation. Alkaline phosphatase, placental-like 2 (ALPPL, RC204330), was obtained as a C-terminal Myc-DDK (FLAG)-tagged ORF clone with the transfection-ready DNA from ORIGENE.

ALPL and ALPP were expressed as fusion proteins with an N-terminal FLAG tag, and ALPI was expressed as a fusion protein of a C-terminal FLAG tag. ALPPL was expressed as a fusion protein with a C-terminal Myc-FLAG-tag. LPP1, LPP2, LPP3, SPP1, and SPP2 were expressed as fusion proteins with a C-terminal green fluorescence protein (GFP) tag. All proteins were expressed in human embryonic kidney 293-F cells (Invitrogen, Carlsbad, CA) with 293 fectin (Invitrogen) according to the manufacturer's protocol. The transfected cells were cultured for 72 h in FreeStyle 293 Expression Medium (Invitrogen). The cells were collected by centrifugation and suspended in 10 mm HEPES, pH 7.0 containing, 0.25 m sucrose, 1 mm DTT, and protease inhibitor mixture (Complete, Roche). After sonication, the suspensions were used as an extract.

Protein expression of ALPL, ALPI, ALPP, and ALPPL was confirmed and quantified by Western blot analysis. Proteins were loaded onto SDS-PAGE gels (4–15% gradient) and then electrotransferred onto a polyvinylidene difluoride membrane by iBlot (Invitrogen). After blocking with AQUA Block (East Coast Biologicals), the membrane was incubated with an anti-FLAG M2 antibody conjugated with horse radish peroxidase (Sigma Aldrich), with subsequent visualization by an ECL Western blotting detection system (GE Healthcare). The band intensity was scanned by a NightOWL imaging system (Berthold technologies, Bad Wildbad, Germany) and quantified using C-terminal FLAG-bacterial alkaline phosphatase (Sigma Aldrich) as a standard protein. Expressed ALPs were confirmed to have phosphatase activity by using a standard substrate of *p*-nitrophenylphosphate (data not shown).

Protein expression of LPP1, LPP2, LPP3, SPP1, and SPP2 was confirmed and quantified by C terminus fused GFP fluorescence. The cells transfected with these genes were plated at a density of 3 × 10^4^ cells/well in a 96-well culture plate. Bisbenzimide H 33342 trihydrochloride (Hoechst 33342) DNA dye (Sigma) was used for staining nuclei at a final concentration of 0.01 μg/ml. GFP fluorescence of these proteins and the nuclei of all cells were visualized (magnification, 40×) with ImageXpress confocal microscopy (Molecular Devices, Sunnyvale, CA), and analyzed using MetaXpress software (Molecular Devices).

##### Immunodepletion of ALPL in Human Kidney Extract

DM was added to the human kidney extract at a final concentration of 0.1%, and rotated at 4 °C for 30 min. Thereafter, the extract was diluted 10-fold with TBS. Monoclonal mouse anti-ALPL antibody (sc-21708, Santa Cruz Biotechnology) or normal mouse IgG was serially diluted with PBS containing 1 mg/ml BSA. The diluted human kidney extract was incubated at 4 °C for 2 h with the diluted anti-ALPL antibody and Protein G agarose (ImmunoPure Plus, PIERCE) in TBS containing 200 μg/ml BSA. Subsequently, the mixture was filtrated using a 0.45 μm spin top filter (Ultrafree MC, Millipore) to remove Protein G agarose and the filtrate was tested for CS-0777-P phosphatase activity. The activity was normalized by the sample diluted by the same factor without immunodepletion.

## RESULTS

### 

#### 

##### Fractionation and Identification of ALPL as a CS-0777-P Phosphatase Candidate

To identify the unknown phosphatase or phosphatases capable of dephosphorylating CS-0777-P in humans, we used protein correlation profiling to identify proteins that co-purified with CS-0777-P dephosphorylation activity on fractionation. At the outset of our search, it was difficult to ascertain which organs or tissues *in vivo* are mainly responsible for the reaction. Therefore, our strategy was first to purify an enzyme from the organ with the highest enzyme activity and identify it; second, we examined the contribution of the enzyme in the organ; third, we examined the contribution of the enzyme in the whole body. We first tested CS-0777-P phosphatase activity in the human lung, kidney, liver, and small intestine to select an appropriate starting material and found equivalent activity in these tissues (data not shown). To choose one tissue among these, we then investigated the tissues of rats and monkeys, demonstrating that the kidney had the highest activity in both species (data not shown). Therefore, we decided to use the human kidney as a purification source for this study.

**Fig. 2.**
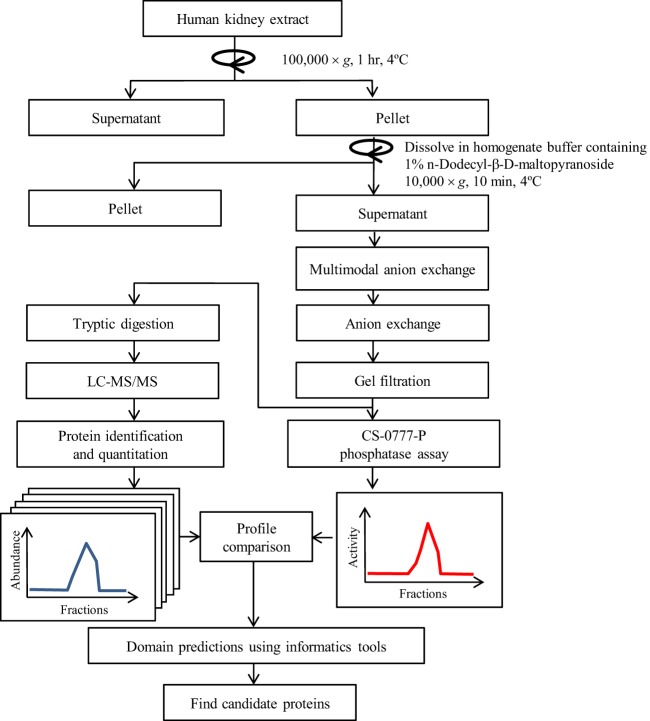
**A schematic diagram of the fraction and proteomic correlation profiling.**

The schematic diagram of this study is indicated in Fig. 2. First, we investigated the CS-0777-P phosphatase activity of kidney extract from five human specimens to assess individual variability and chose the most specimen exhibiting highest levels of enzyme activity as starting material for purification. As shown in supplemental Fig. S1, specimen #5 showed the strongest activity and thus was chosen for purification. Then, we examined biochemical characteristics for CS-0777-P phosphatase activity of the specimen, and found that; 1) the activity mainly resided in the insoluble fraction recovered after ultracentrifugation of human kidney homogenate; 2) the detergent DM effectively re-solubilized the activity; 3) activity was inactivated by EDTA, and then recovered by the addition of Ca^2+^, Mg^2+^, 4) the activity was stable from pH 5 to pH 9, 5) the observed molecular weight of the activity was 150–170 kDa as determined by gel filtration chromatography, 6) the activity was bound to a multimodal anion exchange column (Capto adhere) at pH 9 and an anion exchange column at pH 9, but was not bound to a blue dye column, hydrophobic interaction column, cation exchange column, or a hydroxyl apatite column.

**Fig. 3.**
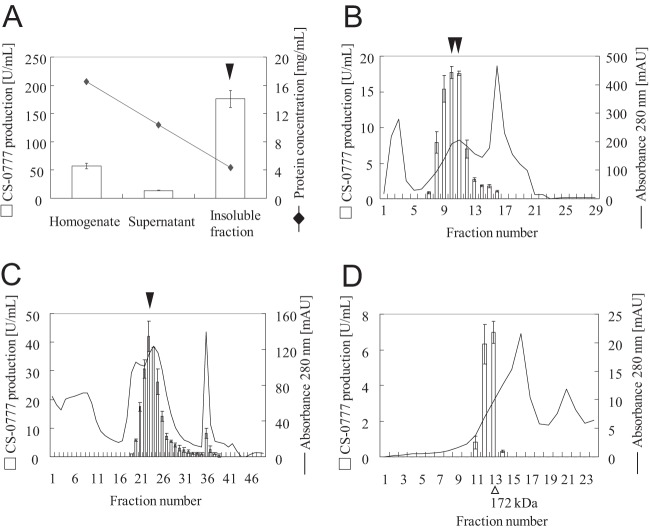
**Purification of CS-0777-P phosphatase in human kidney.** Each fraction was assayed for CS-0777-P phosphatase activity and is shown as bars (left *y* axis). Protein concentration or UV [280 nm] are shown as lines (right *y* axis). *A*, The activity was found in 100,000 × *g* pellet solubilized with 1% DM. Graphs (*B*), (*C*), and (*D*) represent CS-0777-P phosphatase activity and protein concentration in the purification step of the first column (multimodal anion exchange chromatography) (*B*), the second column (anion exchange column) (*C*), and the third column (Gel filtration chromatography) (*D*). CS-0777-P phosphatase activity and protein concentration are indicated by the bars and solid lines, respectively. CS-0777-P phosphatase activity is shown as the average of duplicates with error bars to the minimum and maximum values (*n* = 2). ▾: These fraction samples were used for purification of the next step.

**Fig. 4.**
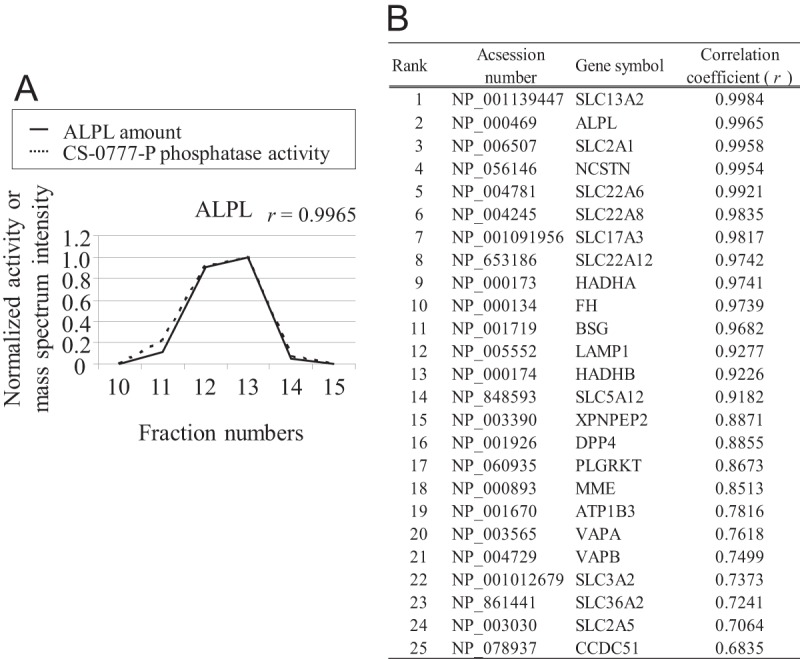
**Correlation between CS-0777-P phosphatase activity and mass spectrometry-based quantification of ALPL.** *A*, The CS-0777-P phosphatase activity and ALPL amount for each fraction. The correlation coefficient between these was 0.9965. ALPL amount and CS-0777-P phosphatase activity are indicated by a solid line and a dashed line, respectively. *B*, Top 25 proteins demonstrating high correlation coefficient between CS-0777-P phosphatase activity and mass spectrometry-based quantitation among the identified 266 proteins. ALPL ranked 2nd place.

We fractionated CS-0777-P phosphatase activity from 1.1 g of human kidney based on the characteristics of the enzyme described above. Because the phosphatase was in insoluble fraction, the purification was very difficult. Despite four successive steps of purification, the enzyme activity was concentrated by only 200-fold (Table I, Fig. 3*A*–3*D*) and we still could not locate a distinct band correlating with CS-0777-P phosphatase activity in the fractions of the final gel filtration chromatography step on SDS-PAGE gel because there were too many other bands on the gel. Thus, we applied proteomic correlation profiling to identify candidate proteins that co-purified with CS-0777-P phosphatase activity. During a proteomic correlation profiling experiment, which assumes that protein quantity correlates with protein activity, all of the samples (chromatographic fractions in this case) are subjected to proteomic analysis to quantify all proteins in parallel with a biological assay (CS-0777-P phosphatase activity). A protein that highly correlates with the desired enzyme activity suggests that the protein could be responsible for the biological activity. To quantify all proteins whose abundance profiles might correlate with CS-0777-P dephosphorylation, all fractions exhibiting target enzyme activity following gel filtration chromatography and several earlier steps of fractionation (Fig. 3*D*) were spiked with an equal amounts of BSA as the internal standard and digested by trypsin prior to LC-MS/MS analysis. Although labeling-based methods provide more accurate quantitation, they also require additional experimental steps and expensive reagent; thus, we chose label-free quantitation for its simplicity, requiring no additional experimental effort or expensive reagents. The proteins were identified and quantified by MaxQuant using an intensity-based label-free algorithm (supplemental Tables S2–S5) (33, 34, 38). After normalization by BSA, the Pearson correlation coefficients were calculated comparing CS-0777-P phosphatase activity levels with protein abundance profiles for each of the 266 identified proteins (supplemental Table S1). The highly correlated proteins were then manually evaluated based on their annotations, expression profiles, and domain structures to identify those most likely to possess the target enzyme activity. Among the top 25 most correlated proteins, ALPL was the only candidate that had a possible phosphatase domain and high correlation efficient of *r* = 0.9965, ranked as 2nd place among all 266 proteins (Fig. 4).

**Table I:** Purification table of CS-0777-P phosphatase from the human kidney

Step	Protein conc. [μg/ml]	Activity [U/ml]	Volume [ml]	Total protein [mg]	Total activity [U]	Specific content [U/mg]	Step fold change	Overall fold change	Step recovery [%]	Overall recovery [%]
Human kidney homogenate	16543	57	10	165	570	3.4	-	1	-	100
1) Centrifugation	4339	176	10	43	1760	41	11.8	12	309	309
2) Multimodal anion exchange (Capto adhere)	188	18	5	0.9	90	96	2.4	28	5.1	16
3) Anion Exchange (Mono Q)	101	42	1	0.1	42	416	4.3	121	47	7.4
4) Gel filtration (Superdex 200)	10	7	0.05	0.0005	0.4	711	1.7	206	0.8	0.1

##### Recombinant Protein Preparation of Candidate Proteins for CS-0777-P Phosphatase

To determine whether ALPL did in fact possess CS-0777-P phosphatase activity, we prepared a recombinant ALPL protein expressed in a mammalian over-expression system. In addition to ALPL, we prepared three other ALP isozymes, *i.e.* ALPI, ALPP and ALPPL, to confirm whether other ALP isoforms could dephosphorylate CS-0777-P. Furthermore, because fingolimod phosphate (fingolimod-P) was reported to be dephosphorylated by LPP3 (30), we also prepared recombinant LPP3 and other proteins close to LPP3 (LPP1, LPP2, SPP1, and SPP2) based on high amino acid sequence homology to LPP3 (39, 40) to investigate their CS-0777-P phosphatase activity.

The expression vector was transfected into a human cell line, 293-F cells. The transfected cells were homogenized and tested for CS-0777-P phosphatase activity. To estimate the expression level of the proteins, the extracts were analyzed by Western blot using an anti-FLAG antibody. Because FLAG-fused proteins migrated poorly on an SDS-PAGE gel, LPP and SPP proteins were instead quantified by GFP fluorescence using confocal microscopy (data not shown).

**Fig. 5.**
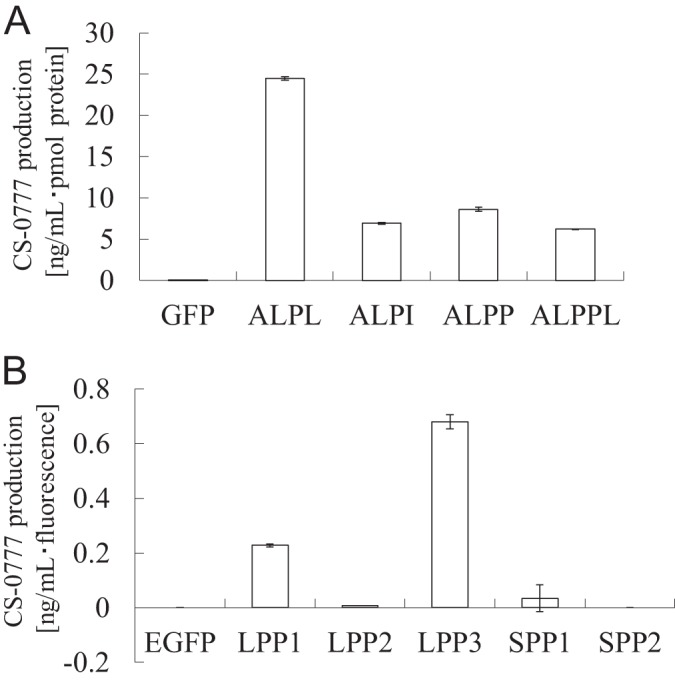
**CS-0777-P phosphatase activity of recombinant proteins.** *A*, Specific CS-0777-P phosphatase activity of the recombinant proteins. Candidate proteins (ALPL, ALPI, ALPP, and ALPPL) and the negative control (EGFP) were expressed in the mammalian cell line, and the enzyme activity was tested. The recombinant proteins were quantified by Western blotting. The data are shown as the average of duplicates with error bars indicating the minimum and maximum values (*n* = 2). *B*, Specific CS-0777-P phosphatase activity of the recombinant proteins. Candidate proteins (LPP1, LPP2, LPP3, SPP1, and SPP2) and the negative control (EGFP) were expressed in the mammalian cell line, and the enzyme activity was tested. The recombinant proteins were quantified by GFP fluorescence. The data are shown as the average of duplicates with error bars representing the minimum and maximum values (*n* = 2).

All four ALP isozymes showed CS-0777-P phosphatase activity (Fig. 5*A*). In particular, ALPL, which was identified from human kidney, had 3-fold higher specific activity compared with the other three ALP isozymes (Fig. 5A). Among the LPP and SPP proteins, only LPP3 and LPP1 showed activity, and the LPP3 had threefold higher specific activity compared with LPP1 (Fig. 5*B*).

##### Effects of ALPL Specific Inhibitor and ALPL Immunodepletion

Although recombinant LPP3 and LPP1 showed CS-0777-P phosphatase activity, we could not identify LPP3 in the purification process. Thus, to determine whether ALPL would be the major CS-0777-P phosphatase candidate in the human kidney, we examined the effects of treatment with an ALPL-specific inhibitor, levamisole (36, 37) and immunodepletion using an anti-human ALPL antibody in the kidney extract.

**Fig. 6.**
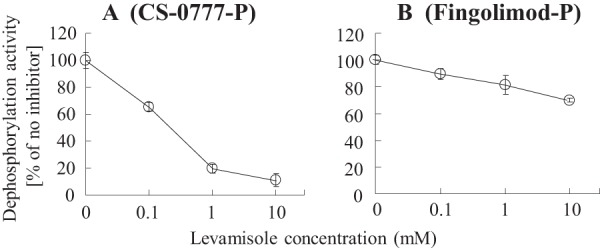
**Effect of the ALPL specific inhibitor on human kidney extract.** The effect of the ALPL specific inhibitor (levamisole) was examined for phosphatase activity from human kidney extract (#5) using CS-0777-P (*A*) or fingolimod-P (*B*) as a substrate. Dephosphorylation activity was normalized by the activity without the inhibitor. The results are expressed as the average of duplicates with error bars to the minimum and maximum values (*n* = 2).

To confirm the specificity of levamisole, we studied its effect on CS-0777-P phosphatase activity of recombinant ALP proteins, LPP1, and LPP3. Among these, levamisole prominently inhibited only CS-0777-P phosphatase activity of ALPL (supplemental Fig. S2). We then examined the effect of levamisole on CS-0777-P phosphatase or fingolimod-P phosphatase activity from the human kidney extract using specimen #5, which was used as a purification source. We found that levamisole inhibited CS-0777-P phosphatase activity in a concentration-dependent manner, and that 1 mm of levamisole inhibited ∼80% of the activity in the extract. This indicated that ALPL, but no other ALPs or LPP proteins, was mainly responsible for the activity (Fig. 6*A*). On the other hand, 10 mm of levamisole inhibited ∼30% of fingolimod-P phosphatase activity in the extract. This demonstrated that ALPL was not responsible for the majority of fingolimod-P phosphatase activity in human kidney extract (Fig. 6*B*).

Next, to confirm the general contribution of ALPL, the effect of levamisole was examined on the other human kidney specimens. More than 50% inhibition by 1 mm levamisole in all the tested samples (supplemental Fig. S3) suggested that ALPL was the major CS-0777-P phosphatase in humans.

**Fig. 7.**
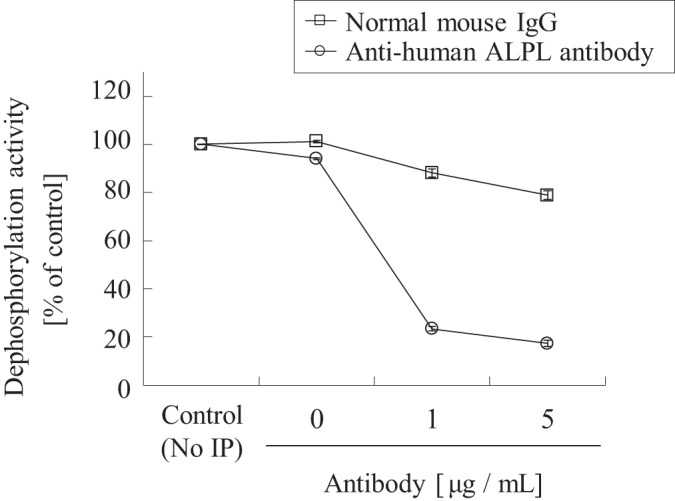
**Immunodepletion of ALPL in human kidney extract.** Human kidney extract was immunodepleted with an anti-human ALPL antibody or normal mouse IgG, and tested for CS-0777-P phosphatase activity. The activity was normalized by the activity of each sample diluted to the same factor without immunodepletion (No-IP). The data are shown as the average of duplicates with error bars to the minimum and maximum values (*n* = 2).

As an independent confirmation, we further attempted immunodepletion experiments using an anti-human ALPL antibody. The human kidney extract was incubated with an anti-human ALPL monoclonal antibody or anti-mouse IgG as a negative control in the presence of protein G-agarose, and the immunodepleted samples were tested for CS-0777-P phosphatase activity. More than 80% of CS-0777-P phosphatase activity in the human kidney extract was immunodepleted with anti-human ALPL antibody (Fig. 7). We confirmed specificity of ALPL antibody by immunodepletion experiments toward recombinant ALPs and LPPs (supplemental Fig. S4). Although the activity of ALPI and ALPPL was removed about 20% by anti-ALPL antibody, the expression of these ALP isozymes was very low in human kidney (data not shown), and thus, the decrease in CS-0777-P phosphatase activity would be due to the results of ALPL depletion from human kidney extract.

To estimate the contribution of the ALPL *in vivo*, we tested levamisole on human liver, lung and small intestine extracts or S9 fraction (the supernatant of tissue extract, see details under “Experimental Procedures”) (supplemental Fig. S5). Levamisole inhibited the dephosphorylation activity in the liver and lung, suggesting that the major enzyme in those tissues would be ALPL. We also examined levamisole on the dephosphorylation of CS-0777-P *in vivo* using rats to detect the inhibitory effect. Unfortunately, levamisole had little influence on the blood concentration of CS-0777 in rats after administration of CS-0777-P due to the insufficient exposure of levamisole even at the maximum tolerant dose (50 mg/kg s.c.).

Taken together, all of the data consistently support that ALPL is the major CS-0777-P phosphatase in human kidneys, whereas the contribution of other ALP proteins, LPP1 and LPP3 is low.

## DISCUSSION

In this study, we have successfully purified and identified the primary phosphatase candidate responsible for dephosphorylation CS-0777-P in human kidneys by improved proteomic correlation profiling, an integration of traditional biochemistry and state-of-the-art proteomics technology.

The identified CS-0777-P phosphatase, ALPL, was clearly distinguishable from the reported fingolimod-P phosphatase, LPP3. In addition, we have revealed that ALPL is the major CS-0777-P phosphatase candidate for the human kidney, and that the contribution was estimated to be more than 80% based on the following three observations; 1) we observed a single broad active peak in all purification steps (Fig. 3); 2) levamisole as an ALPL specific inhibitor inhibited CS-0777-P phosphatase activity in a concentration-dependent manner, with 1 mm of levamisole inhibiting ∼80% of the activity in human kidney extract (Fig. 6); and 3) immunodepletion using an anti-human ALPL antibody removed more than 80% of CS-0777-P phosphatase activity in human kidney extract (Fig. 7).

We have demonstrated that the phosphatases of fingolimod-P and CS-0777-P were different enzymes. Given this observation, it is intriguing that identified kinases for fingolimod and CS-0777 were also different (6, 28). That different enzymes are responsible for catalyzing phosphorylation and de-phosphorylation of these related molecules could have important implications for drug metabolism, as each could thus be subject to distinct pharmacological effects (*e.g.* extensive metabolizer, poor metabolizer), and could be associated with different safety profiles and administration requirements (*e.g.* dosing). Indeed, the concentration of CS-0777-P, an active metabolite, in the phase I study of healthy volunteers was more than 20-fold higher than CS-0777 (26), whereas the concentration of fingolimod-P was about the same concentration of fingolimod (41), highlighting these metabolic differences. Thus, in addition to the selectivity, inefficient dephosphorylation of CS-0777-P could demonstrate a better pharmacological benefit than fingolimod-P. Although contributions of the other ALP isozymes *in vivo* need to be clarified, identification of CS-0777-P phosphatase in the kidney represents a critical first step to fully understand the pharmacokinetics and pharmacodynamics of CS-0777.

Purification of a biologically active protein from complex endogenous biological sources is a challenging task, usually requiring large amounts of samples and many separation steps. Traditionally, to identify an enzyme with some desired activity, the responsible protein had to be isolated at sufficient quantity and purity to enable its correlation with enzyme activity by following a distinct band on the SDS-PAGE gel; such an approach was both time-consuming and labor-intensive. Moreover, human ALPs are membrane-bound enzymes (42). Membrane proteins are known to be difficult to purify because solubilization is necessary before chromatography, the most common technique for protein purification. Indeed, in previous reports, ALPs were purified from very large amounts of starting materials, such as 500 g of human placentas with six steps (43), 21 g of human placenta with between seven to nine steps (44), or 1–3 kg of bovine kidney with six steps (45). In this study, ALPL was identified as the active enzyme from only about 1 g of human kidney with four purification steps, which clearly demonstrated the advantages of proteomic correlation profiling.

In proteomic correlation profiling, we first calculated each protein's correlation coefficient between the enzyme activity profile and protein abundance profile determined by proteomics technology toward chromatography fractions. Then, we could select possible candidates for the enzyme among proteins with a high correlation value by domain predictions using informatics tools. By using this technology, we do not have to observe the separated bands correlating with enzyme activity; thereby, reducing the purification steps and starting materials because the required purity is much less than conventional approaches. Indeed, we could identify the ALPL as a candidate protein from only a 200-fold purification of human kidney extract in this study, although we were not able to confirm the enzyme activity solely on the basis of correlation, as there were numerous other species on the band of the SDS-PAGE gel that correlated with the enzyme activity by this 4-step purification (supplemental Fig. S6). Furthermore, we retrospectively applied proteomic correlation profiling in the third step fraction, anion exchange chromatography, and found that ALPL was identified as the highest ranked protein having a phosphatase domain while only one another protein had the domain. This indicated that we could have reduced one purification step further to identify ALPL (supplemental Fig. S7); however, there are limitations to this method. 1) If post-translational modification regulates an enzyme activity and the profile of the modified protein is different from that of the total protein, the method should not work because current proteomic correlation profiling is based on the profile of total proteins. 2) If there is an endogenous inhibitor protein in the fractions, the enzyme activity profile should not correlate with the simple protein amount of a single protein. 3) If more than one protein are responsible for the targeted enzyme activity and the profiles of those proteins are not separated in chromatography, again the enzyme activity profile should not correlate with the amount of a single protein. In addition, 4) if we cannot choose candidate proteins based on protein domain information, it is highly possible that we have too many candidates. In all cases above, adding additional purification steps and incorporating other sources of biological information could further reduce the list of candidates to a more manageable number.

In this study, we enhanced the protein correlation profiling approach from our previous study by incorporating label-free intensity-based protein quantitation as implemented in the recently developed MaxQuant software suite (33, 34, 38). We introduced a protein internal standard into each fraction to normalize for variations in sample complexity among fractions. It is noteworthy that this normalization strategy was critical for successful identification of the protein responsible for our desired activity. This is the first study using this technology for the enzyme class other than kinase, and moreover, the first example using one of the most complex and notorious protein mixtures, a tissue extract as a purification source. We believe that this approach can be further extended well beyond kinases and phosphatases to a wide variety of enzyme activities, and that this study in particular establishes a foundation for proteomic correlation profiling to be used as a general method.

## Supplementary Material

Supplemental Data

## Footnotes

^1^ The abbreviations used are:

S1P1: sphingosine 1-phosphate receptor 1
ALPL: alkaline phosphatase, tissue-nonspecific isozyme
ALPI: alkaline phosphatase, intestinal
ALPP: alkaline phosphatase, placental
ALPPL: alkaline phosphatase, placental-like 2
DM: n-Dodecyl-β-D-maltopyranoside
LPP1: lipid phosphate phosphohydrolase 1
LPP2: lipid phosphate phosphohydrolase 2
LPP3: lipid phosphate phosphohydrolase 3
SPP1: sphingosine-1-phosphate phosphatase 1
SPP2: sphingosine-1-phosphate phosphatase 2
FN3K: fructosamine 3-kinase
FN3K-RP: fructosamine 3-kinase-related protein
HEPES: 4-(2-hydroxyethyl)-1-piperazineethanesulfonic acid
LC-MS/MS: liquid chromatography equipped with tandem mass spectrometry
SDS-PAGE: sodium dodecyl sulfate-polyacrylamide gel electrophoresis
S1P: sphingosine 1-phosphate
S1P3: sphingosine 1-phosphate receptor 3
SPHK1: sphingosine kinase 1
SPHK2: sphingosine kinase 2.
