# Supplementary material for: Identification of a Metabolizing Enzyme in Human Kidney by Proteomic Correlation Profiling
Source: Mol Cell Proteomics. 2013 May 14;12(8):2313–23. doi: 10.1074/mcp.M112.023853 (PMC3734587; doi:10.1074/mcp.M112.023853)
Supplement: Supplemental Data [file supp_12_8_2313__index.html]

Identification of a metabolizing enzyme in human kidney by proteomic correlation profiling — Identification of a Metabolizing Enzyme in Human Kidney by Proteomic Correlation Profiling — Enzyme Identification by Proteomic Correlation Profiling — Supplemental Data 

# Identification of a Metabolizing Enzyme in Human Kidney by Proteomic Correlation Profiling

## Supplemental Data

**Files in this Data Supplement:**

- Supplemental figures - Supplemental figures
- Supplemental tables - Supplemental tables
