# Supplementary material for: Identification of a Metabolizing Enzyme in Human Kidney by Proteomic Correlation Profiling
Source: Mol Cell Proteomics. 2013 May 14;12(8):2313–23. doi: 10.1074/mcp.M112.023853 (PMC3734587; doi:10.1074/mcp.M112.023853)
Supplement: Supplemental Data [file supp_M112.023853_mcp.M112.023853-1.pptx]

## Slide 1
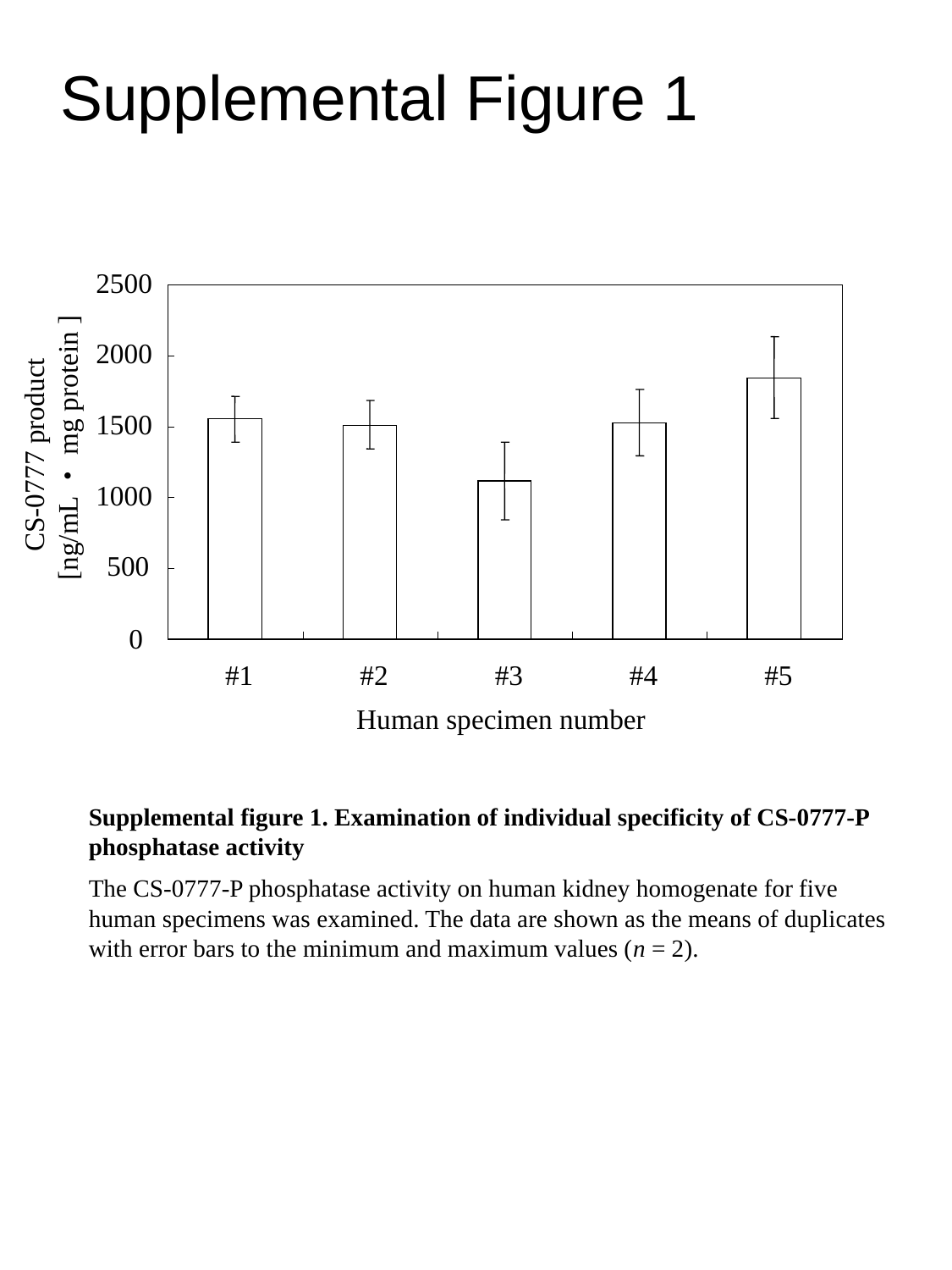

# Supplemental Figure 1
2500
2000
1500
1000
500
CS-0777 product
 [ng/mL ･ mg protein ]
0
#1
#2
#3
#4
#5
Human specimen number
Supplemental figure 1. Examination of individual specificity of CS-0777-P phosphatase activity
The CS-0777-P phosphatase activity on human kidney homogenate for five human specimens was examined. The data are shown as the means of duplicates with error bars to the minimum and maximum values (n = 2).

## Slide 2
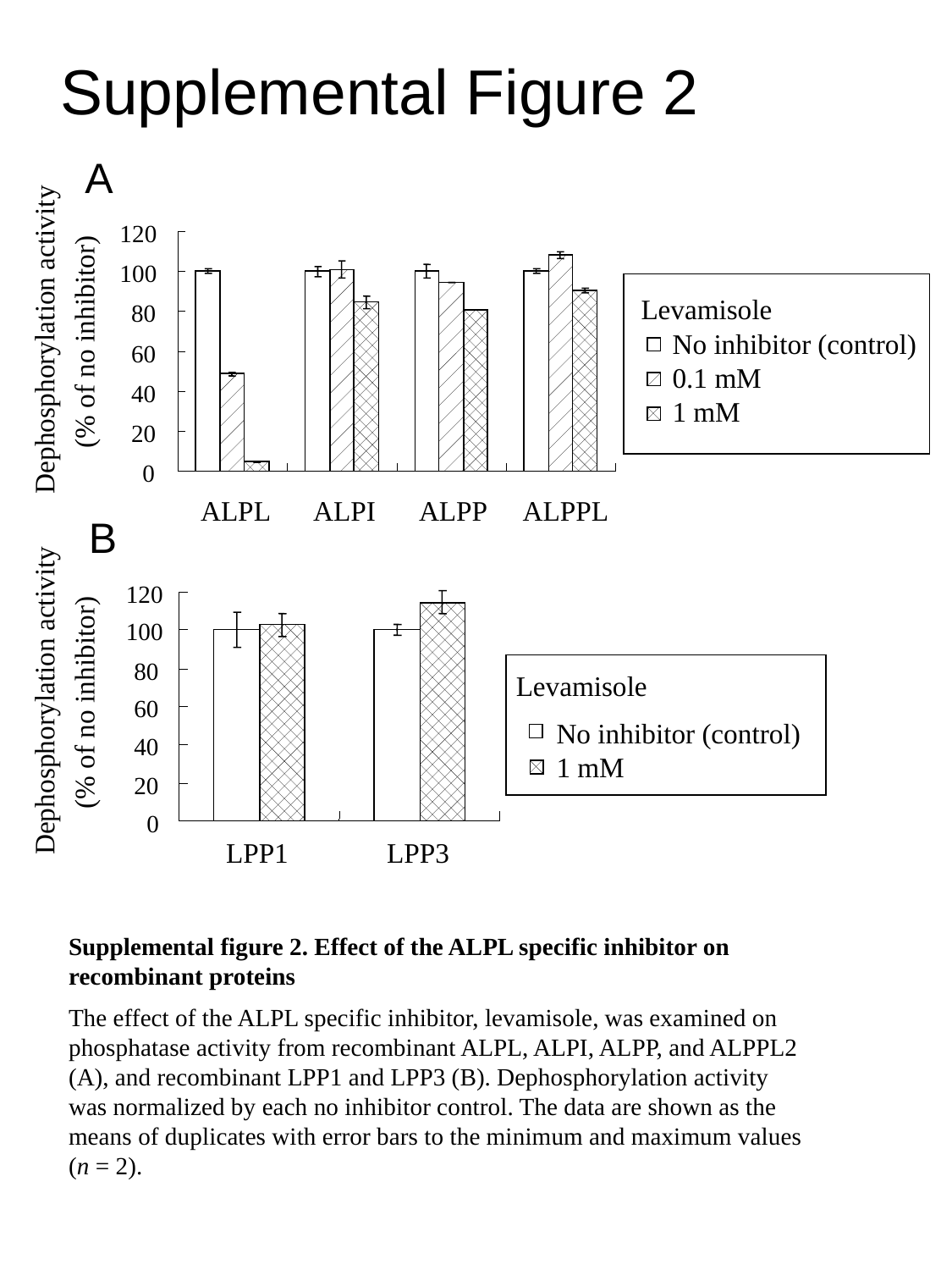

# Supplemental Figure 2
A
120
100
Levamisole
80
Dephosphorylation activity
No inhibitor (control)
0.1 mM
1 mM
 (% of no inhibitor)
60
40
20
0
ALPL
ALPI
ALPP
ALPPL
B
120
100
80
Levamisole
Dephosphorylation activity
 (% of no inhibitor)
60
No inhibitor (control)
1 mM
40
20
0
LPP1
LPP3
Supplemental figure 2. Effect of the ALPL specific inhibitor on recombinant proteins
The effect of the ALPL specific inhibitor, levamisole, was examined on phosphatase activity from recombinant ALPL, ALPI, ALPP, and ALPPL2 (A), and recombinant LPP1 and LPP3 (B). Dephosphorylation activity was normalized by each no inhibitor control. The data are shown as the means of duplicates with error bars to the minimum and maximum values (n = 2).

## Slide 3
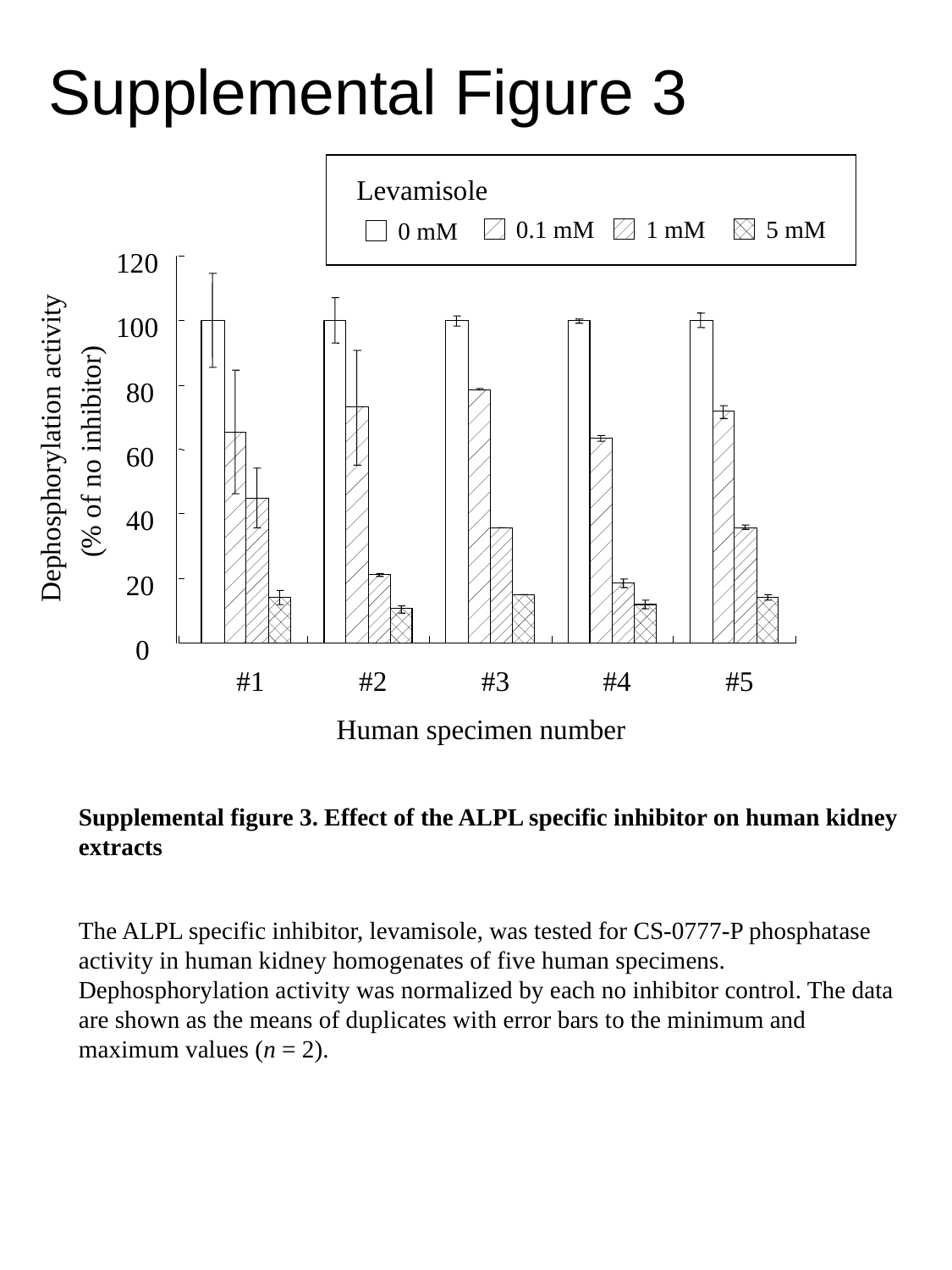

# Supplemental Figure 3
Levamisole
0.1 mM
1 mM
5 mM
0 mM
120
100
80
Dephosphorylation activity
 (% of no inhibitor)
60
40
20
0
#2
#3
#4
#5
#1
Human specimen number
Supplemental figure 3. Effect of the ALPL specific inhibitor on human kidney extracts
The ALPL specific inhibitor, levamisole, was tested for CS-0777-P phosphatase activity in human kidney homogenates of five human specimens. Dephosphorylation activity was normalized by each no inhibitor control. The data are shown as the means of duplicates with error bars to the minimum and maximum values (n = 2).

## Slide 4
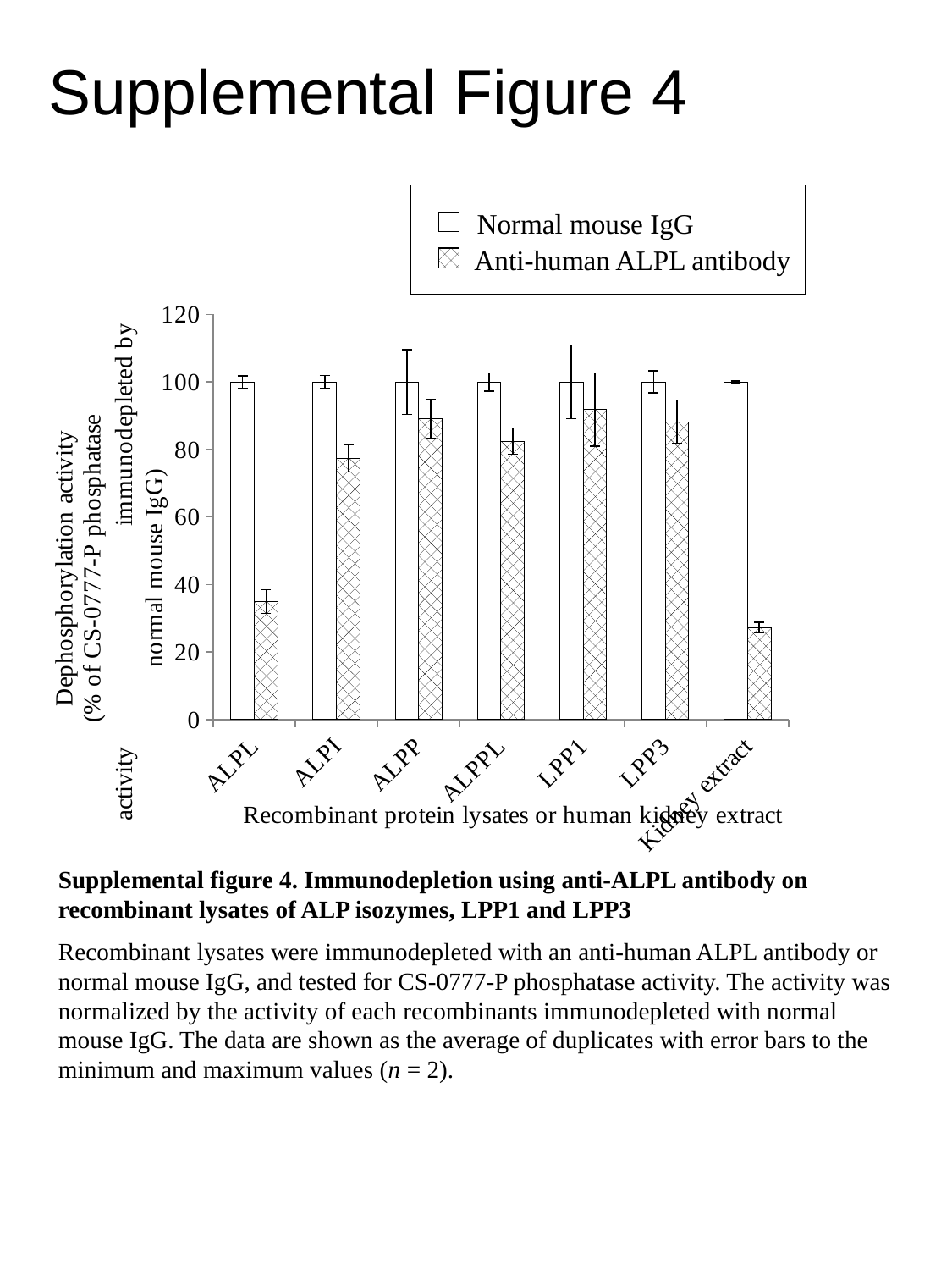

Supplemental Figure 4
Normal mouse IgG
### Chart
| Category | Normal mouse IgG 　　as negative control | Anti-ALPL antibody [final conc. 5 ug/mL] |
|---|---|---|
| ALPL | 100.0 | 34.95059008760704 |
| ALPI | 100.0 | 77.45601015766258 |
| ALPP | 100.0 | 89.10114656664095 |
| ALPPL | 100.0 | 82.46452740298284 |
| LPP1 | 100.0 | 91.82249120475973 |
| LPP3 | 100.0 | 88.22911204642008 |
| Kidney extract | 100.0 | 27.239025126461854 |Anti-human ALPL antibody
Supplemental figure 4. Immunodepletion using anti-ALPL antibody on recombinant lysates of ALP isozymes, LPP1 and LPP3
Recombinant lysates were immunodepleted with an anti-human ALPL antibody or normal mouse IgG, and tested for CS-0777-P phosphatase activity. The activity was normalized by the activity of each recombinants immunodepleted with normal mouse IgG. The data are shown as the average of duplicates with error bars to the minimum and maximum values (n = 2).

## Slide 5
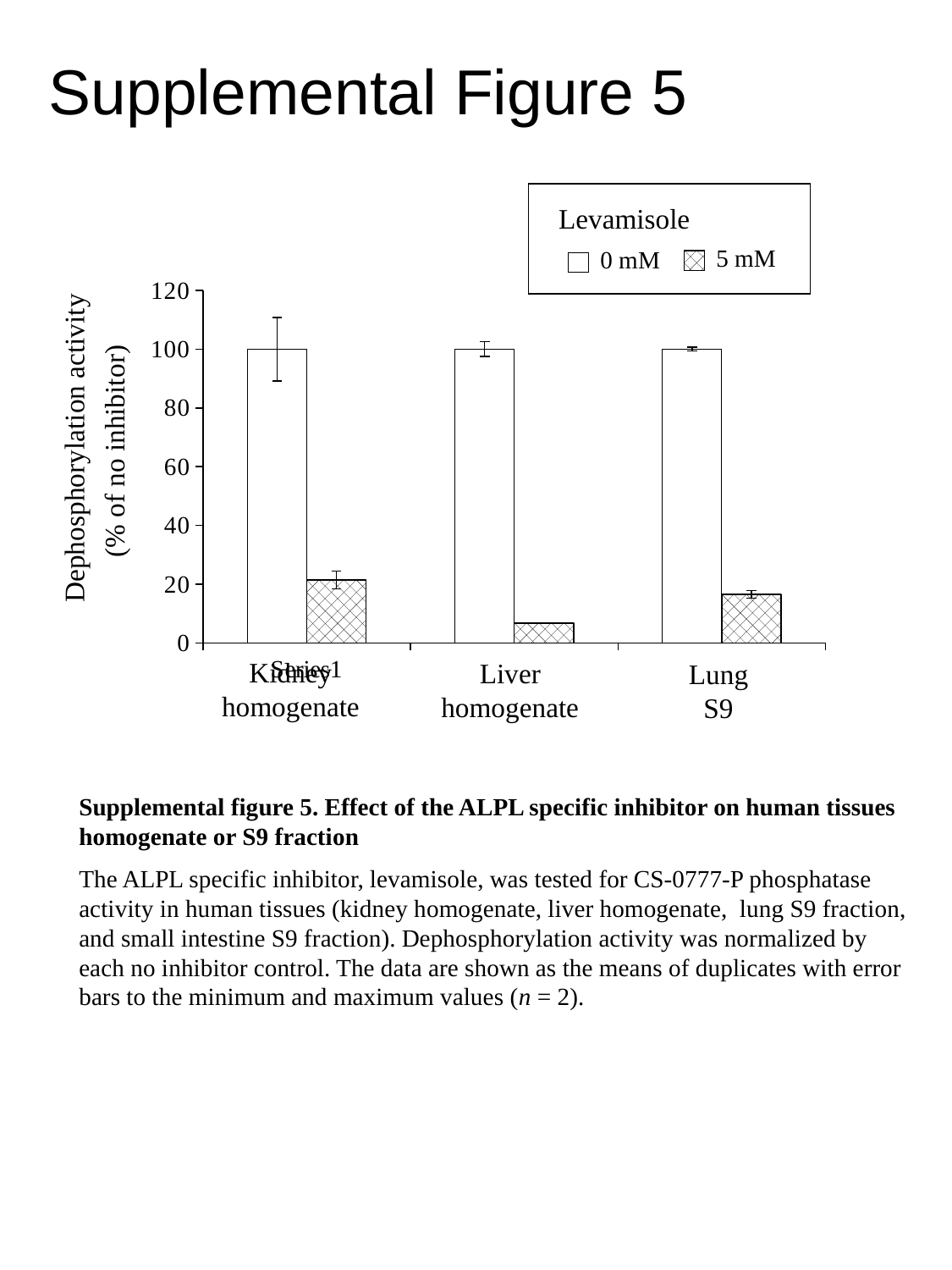

# Supplemental Figure 5
Levamisole
### Chart
| Category | | |
|---|---|---|
| | 100.0 | 21.408860889931447 |
| | 100.0 | 6.820991562652052 |
| | 100.0 | 16.536098870646207 |5 mM
0 mM
Dephosphorylation activity
 (% of no inhibitor)
Kidney
homogenate
Liver
homogenate
Lung
S9
Supplemental figure 5. Effect of the ALPL specific inhibitor on human tissues homogenate or S9 fraction
The ALPL specific inhibitor, levamisole, was tested for CS-0777-P phosphatase activity in human tissues (kidney homogenate, liver homogenate, lung S9 fraction, and small intestine S9 fraction). Dephosphorylation activity was normalized by each no inhibitor control. The data are shown as the means of duplicates with error bars to the minimum and maximum values (n = 2).

## Slide 6
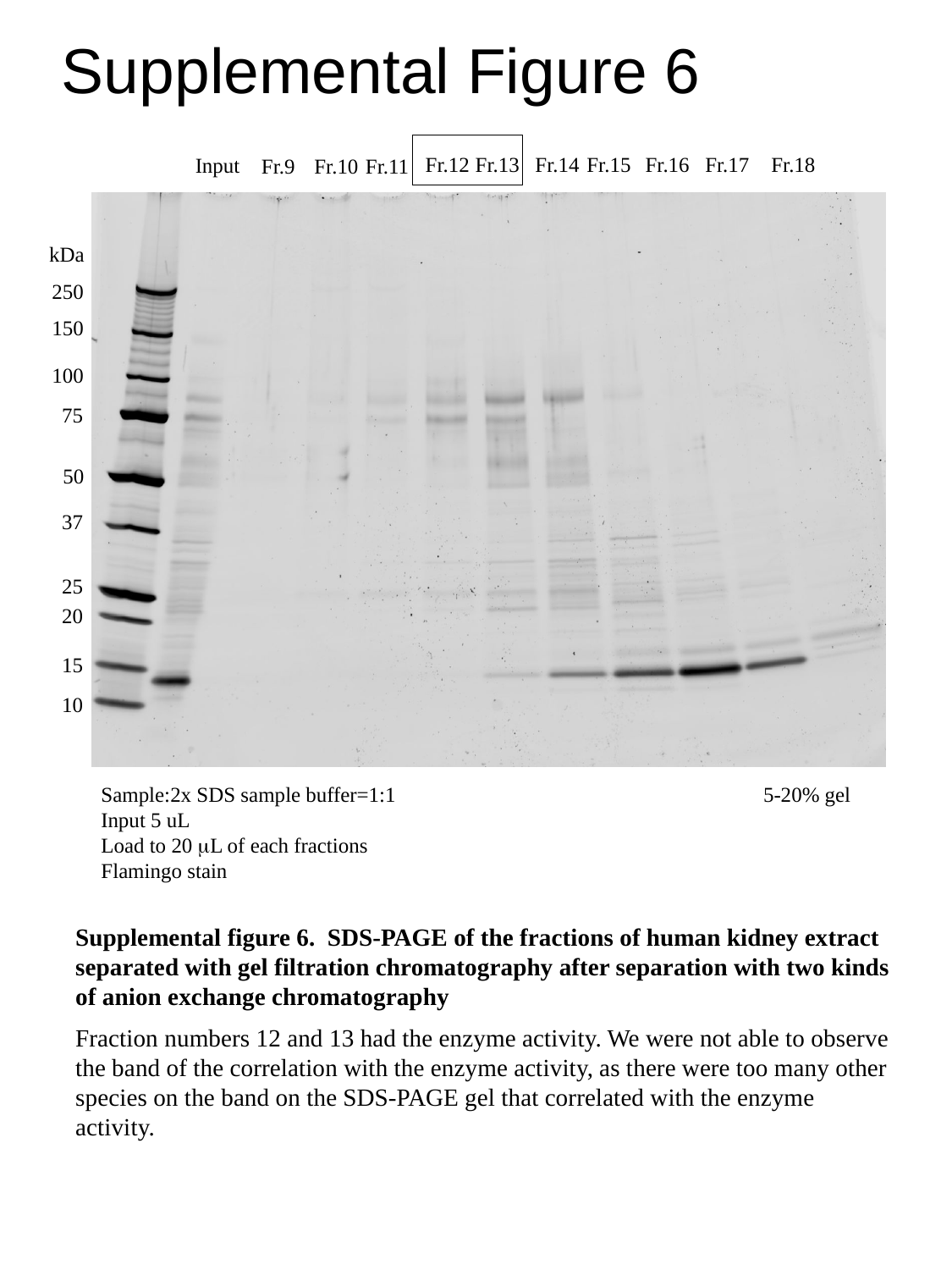

# Supplemental Figure 6
Fr.12
Fr.13
Fr.14
Fr.15
Fr.16
Fr.17
Fr.18
Input
Fr.9
Fr.10
Fr.11
kDa
250
150
100
75
50
37
25
20
15
10
5-20% gel
Sample:2x SDS sample buffer=1:1
Input 5 uL
Load to 20 μL of each fractions
Flamingo stain
Supplemental figure 6. SDS-PAGE of the fractions of human kidney extract separated with gel filtration chromatography after separation with two kinds of anion exchange chromatography
Fraction numbers 12 and 13 had the enzyme activity. We were not able to observe the band of the correlation with the enzyme activity, as there were too many other species on the band on the SDS-PAGE gel that correlated with the enzyme activity.

## Slide 7
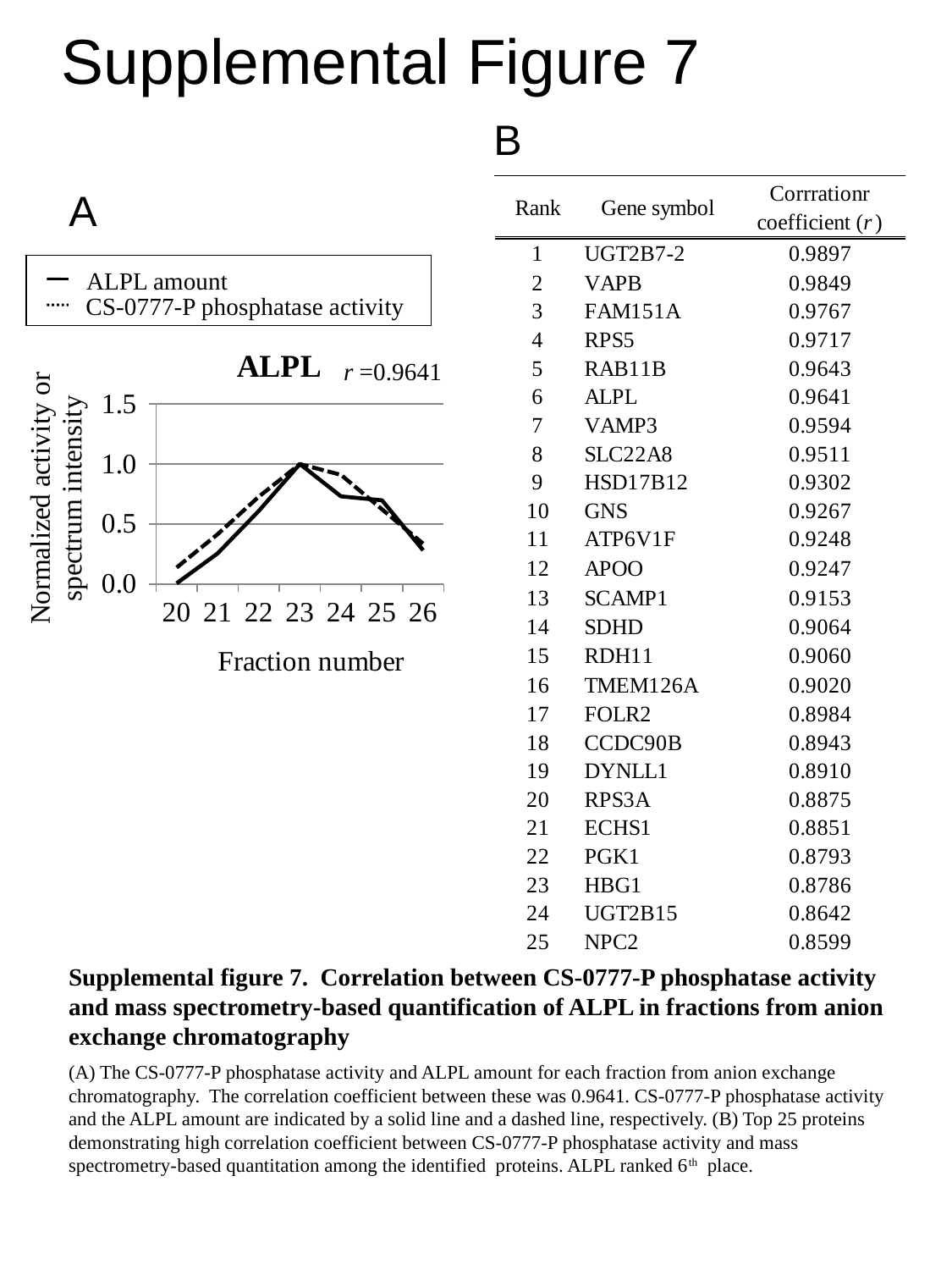

# Supplemental Figure 7
B
A
ALPL amount
CS-0777-P phosphatase activity
### Chart: ALPL
| Category | | |
|---|---|---|
| 20 | 0.13724535233029764 | 0.005631562844006644 |
| 21 | 0.41591867858389353 | 0.25610119609980375 |
| 22 | 0.7276879653134414 | 0.6091946743243348 |
| 23 | 1.0 | 1.0 |
| 24 | 0.9101650595897472 | 0.7305963086636827 |
| 25 | 0.6224734965990524 | 0.6977295992797008 |
| 26 | 0.33637047115390867 | 0.28063435632547046 |r =0.9641
Supplemental figure 7. Correlation between CS-0777-P phosphatase activity and mass spectrometry-based quantification of ALPL in fractions from anion exchange chromatography
(A) The CS-0777-P phosphatase activity and ALPL amount for each fraction from anion exchange chromatography. The correlation coefficient between these was 0.9641. CS-0777-P phosphatase activity and the ALPL amount are indicated by a solid line and a dashed line, respectively. (B) Top 25 proteins demonstrating high correlation coefficient between CS-0777-P phosphatase activity and mass spectrometry-based quantitation among the identified proteins. ALPL ranked 6th place.
